# Optimizing Feeding Schedule and Live-Weight Prediction for Native Chicken Based on Machine Learning

**DOI:** 10.3390/ani16010075

**Published:** 2025-12-26

**Authors:** Chung-Liang Chang, Rui-Yi Xu

**Affiliations:** Department of Biomechatronics Engineering, National Pingtung University of Science and Technology, Neipu, Pingtung 91201, Taiwan

**Keywords:** indigenous chickens, precision feeding, precision nutrition, ration scheduling, data-driven models, feed conversion ratio, agricultural practice

## Abstract

To meet market supply and demand, producers must accurately schedule processing dates to ensure optimal pricing. This study developed a practical feeding program system for local Taiwanese chicken breeds, including Guzao males, Huangjin females, and Red Junglefowl males. The system integrates daily predictions of cage-level body weight to guide each flock toward a target weight before the planned processing date. Four prediction models were evaluated, including random forest, XGBoost, Extra Trees, and an artificial neural network. The best-performing model was embedded into the system, and an Extra Trees model was used to estimate the total remaining ration and update daily feed allocations under standard feeding conditions. A validation experiment was conducted using a 54-day batch of Guzao males, during which cage-level data were collected. The feed conversion ratio of birds managed under the feeding program was compared with that of conventional feeding. The results provide preliminary support for the feasibility of a data-guided feeding program system with potential agricultural application value, although additional batches and cross-farm evaluations are needed to confirm generalizability and operational performance.

## Simple Summary

This study presents a preliminary proof-of-concept feeding program tool for native Taiwanese chickens. The system predicts the body weight of cage-reared birds and recommends daily feed allocations to help them reach predetermined target weights. The results provide an initial validation of the data-driven feeding program concept, indicating potential agricultural applicability. However, additional batches and cross-farm evaluations are required to further assess robustness and practical effectiveness.

## 1. Introduction

Over the past decade, poultry meat has consistently represented a substantial proportion of global meat consumption, and multiple projections indicate continued growth in demand through 2050 [1,2,3]. Precision livestock farming (PLF) has emerged as an important approach to enhance production efficiency and sustainability, with advances in environmental monitoring, health and welfare assessment, and data-driven decision support demonstrating feasibility in commercial poultry houses [4,5,6]. Nonetheless, most existing PLF applications have been developed for intensive production systems, and their implementation in small-scale or native chicken operations remains limited. Addressing this gap provides an opportunity to adapt PLF concepts to alternative production contexts.

Recent advances in PLF have also expanded toward sensor-based ration adjustment and precision-feeding frameworks that modify daily feed allocations in response to environmental load, activity patterns, or short-term deviations from expected growth. These systems have been evaluated primarily in broilers and other intensive production settings, with far fewer applications in native or colored-feather chickens where flock sizes are smaller and growth trajectories exhibit substantial genetic heterogeneity [7,8,9]. The emerging literature indicates that precision feeding can reduce feed waste, improve feed conversion ratio (FCR), and help stabilize target slaughter weights, yet its adoption in indigenous chicken systems remains limited.

Nevertheless, given the diversity of indigenous chicken breeds, the marked variation in genetic lines and breeding objectives, and differences in farm-level management, experience-based feeding alone is insufficient to achieve precise control of growth trajectories across genotypes. Consequently, feed misallocation may fail to meet the actual growth requirements of the birds. Both over- and under-rationing can increase feed waste, elevate the FCR, raise production costs, and shift slaughter age away from the planned schedule, thereby increasing output variability and supply-chain uncertainty. In particular, when feeding strategies are not updated in accordance with physiological rhythms and breed-specific growth patterns, birds may exhibit compensatory growth accompanied by potential health risks [10].

In Taiwan, the production of native or colored-feather chickens constitutes a distinct market segment that serves differentiated fresh-market demand [11]. Commercial lines commonly encountered include Guzao-type males, Huangjin-type females, and red- feather males, which are phenotypically similar to traditional indigenous chicken types and may exhibit Red Junglefowl-like characteristics. These lines differ in growth rate, mature live weight, carcass composition, and eating quality, reflecting long-term selection within red- and black-feather commercial populations [12]. Sectoral summaries identify red-feather and black-feather native chickens, alongside white broilers and black-bone chickens, as principal meat-type categories in Taiwan. Empirical evaluations have quantified growth curves and carcass traits for red-feather commercial roosters under routine husbandry [13], and population-genetic analyses describe the admixed structure of Taiwanese commercial native chickens relative to specialized occidental breeds [14], which together help explain heterogeneity in performance under field conditions. Because products are marketed to specific ages and body-weight ranges, producers routinely schedule slaughter to meet retail demand and quality targets.

Across agriculture and animal production, machine learning (ML) has been increasingly adopted to support prediction, forecasting, and decision support, including time series modeling, multivariate regression, classification, and data fusion from heterogeneous sensors [15,16,17,18,19]. Methods commonly evaluated include linear and tree- based ensembles (for example, random forest (RF), gradient boosting, and XGBoost), nearest neighbor algorithms, and neural networks; these approaches provide complementary strengths in handling nonlinearity, interactions, and noisy field data while enabling model selection based on out-of-sample performance. In poultry-focused applications, support vector machines and random forests have been used to predict disease risk and abnormal behaviors, improving early warning accuracy [20]. In addition, behavioral and feeding event data have been used to predict pig live weight and market class [21], and algorithms such as XGBoost and RF have been applied to forecast growth and FCR [22]. Recent studies have also applied artificial intelligence to assess chicken activity levels [23] and to perform sex identification in chickens [24]. For broilers and indigenous chickens, artificial neural networks (ANN), multivariate adaptive regression splines, and parametric growth functions such as the Gompertz nonlinear growth model offer complementary capabilities. Neural networks and multivariate adaptive regression splines act as flexible, data-driven approximators that can represent localized curvature and context-specific nonlinearities in the weight–age relationship, whereas parametric curves impose a biologically motivated form and return interpretable parameters, including asymptotic weight and maturation rate [25]. Hybrid approaches that fuse data-driven learners with mechanistic growth curves can provide both accuracy and robustness [26,27,28,29]. In parallel, sensor-based control of feeding, which adapts rations over time using environmental and activity data, has been explored to improve FCR and overall growth performance [30,31,32].

Empirical-based rearing methods hinder precise control of target slaughter age, leading to feed waste and increased costs. This study develops a machine learning-based system for predicting live weight and formulating feed in cages. This system combines chicken age, diet records, and environmental measurements, adjusting feed formulations based on breed and age to guide chickens to reach the user-specified target weight before the scheduled slaughter date. The system was evaluated using three native Taiwanese chicken breeds (Guzao males, Huangjin females, and Red Junglefowl males). The goals and contributions of this study are as follows:

1. Establish a standardized process for collecting and processing local chicken data, integrating age, environmental factors, and feed intake into a dataset suitable for modeling.
2. Compare and evaluate supervised machine learning models for live-weight prediction, and provide an interpretable, field-deployable predictive model for local chickens.
3. Design a preliminary proof-of-concept machine learning system for precision-feeding programs. This system aims to synchronize with planned slaughter dates, using closed-loop simulations to adjust predicted growth curves to achieve specific targets and set daily feed allocations.

The remainder of the paper is organized as follows. Section 2 describes the materials and methods, including data collection, preprocessing, four supervised models for body-weight prediction (RF, XGBoost, Extra Trees (ET), ANN), and the ET-based feeding schedule. Section 3 reports predictive performance on held-out test sets stratified by batch, provides breed-specific evaluation metrics with confidence intervals, analyzes feature importance rankings, and presents illustrative feeding schedule outcomes. Section 4 discusses implications and future directions, and the conclusions are presented in Section 5.

## 2. Materials and Methods

**Figure 1:**
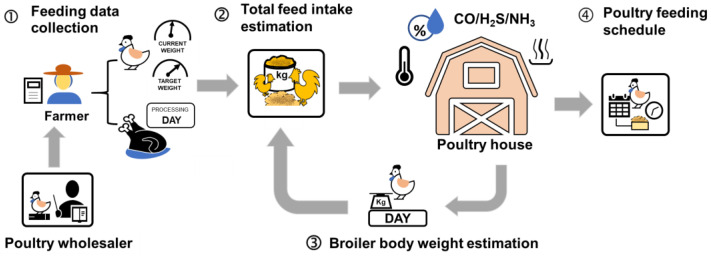
Design concept of poultry feeding schedule.

The design concept of the feeding scheduling system is illustrated in Figure 1. Market demand and planned processing dates are communicated to producers by wholesalers, from which a target live weight and desired processing date are specified. The current flock average live weight is verified, after which the scheduling procedure is initiated. Birds are reared in commercial poultry houses. Given age, target live weight, planned processing date, and environmental conditions, the system estimates the cage level live weight and the required daily ration, and adjusts the daily feed supply according to the observed cage average live weight and cumulative intake. Routine monitoring includes body weight and feed intake measurements, and daily allocations are updated according to system recommendations to guide each cage toward the target live weight on schedule.

Based on this workflow, a two-stage practical feeding scheduling system was adopted. In stage one, predictive models for daily live weight and interval total-feed requirement were developed; in stage two, a daily feeding schedule was generated and iteratively updated using new observations. In the present implementation, the optimization targets the day-to-day allocation of a fixed commercial diet, so nutrient composition was not modified by the feeding planning module.

Let i =1,…,n denote the day index and let g(i) map each day to its stocking barn (batch). The raw date string $t_{i}^{\mathrm{raw}}$ was parsed into ISO-8601 datetime format $t_{i}$ [33], and bird age was computed as the day-level difference between $t_{i}$ and the batch-specific start date $t_{g(i),0}$.

(1) $${\mathrm{Age}}_{i}=\frac{t_{i}-t_{g(i),0}}{1\;\mathrm{day}}\;.$$

Let $N_{i}$ denote the number of birds and $F_{i}$ the total feed provided on day i (g/day); the average daily feed allowance per bird is then

(2) $$f_{i}=\frac{F_{i}}{N_{i}}\;(g/\mathrm{day}),$$

and the cage total weight is recorded as $W_{i}$ (g). During rearing, the index set of environmental records for day i is $D_{i}$; each record $k\;\in \;D_{i}$ contains temperature $T_{i,k}$, relative humidity ${\mathrm{RH}}_{i,k}$, carbon monoxide ${\mathrm{CO}}_{i,k}$, hydrogen sulfide ${H_{2}S}_{i,k}$, and ammonia ${NH_{3}}_{i,k}$. These are aggregated to daily statistics: mean temperature ${\bar{T}}_{i}$ and mean relative humidity ${\bar{\mathrm{RH}}}_{i}$, and daily maxima ${\left(\mathrm{CO}\right)}_{i}^{\max}$, $(H_{2}S{)}_{i}^{\max}$, and $(NH_{3}{)}_{i}^{\max}$.

### 2.1. System Modeling

#### 2.1.1. Daily Weight Prediction Model

The input feature vector for daily live-weight prediction at the cage level includes age, environmental summaries, and the daily ration.

(3) $$x_{i}=[{\mathrm{Age}}_{i},\;{\bar{T}}_{i},\;{\bar{\mathrm{RH}}}_{i},\;f_{i},\;(\mathrm{CO}{)}_{i}^{\max},\;(H_{2}S{)}_{i}^{\max},\;(NH_{3}{)}_{i}^{\max}\;{]}^{⊤}\in \;R^{7}.$$

The regression target is the cage average live weight per bird on day i, $y_{i}=W_{i}/N_{i}$ (g/chicken). Three supervised machine learning models are compared for this task: RF, XGBoost, and ET. RF and ET are ensemble tree methods; RF uses feature and split randomness across trees, whereas ET fully randomizes split thresholds. XGBoost is a boosting framework that adds weak learners to improve predictive accuracy.

#### 2.1.2. Total-Feed Forecasting Model

Total-feed requirements over a growth interval are modeled with intervals indexed by $r\;=\;1,\ldots ,\;n_{b}$. The input vector contains starting live weight, target live weight, and remaining rearing days:

(4) $$x_{a,r}\;=\;[w_{r}^{\mathrm{start}},\;w_{r}^{\mathrm{target}},\;d_{r}^{\mathrm{remain}}{]}^{⊤}\in {\;R}^{3}.$$

where $[.{]}^{⊤}$ denotes the transpose operator. The output is the total feed required for interval r, $y_{b,r}$ (g/chicken over the interval). This model is implemented with ET, as the method handles feature and threshold randomness efficiently and exhibits strong generalization for interval-level total predictions.

### 2.2. Data Preprocessing and Normalization

Environmental and feed records were consolidated to a daily resolution, and calendar dates were converted to rearing age (days). Sensors continuously recorded temperature, relative humidity, CO, H_2_S, and NH_3_. For each rearing day d, environmental summaries were computed over the 24 h window preceding the fixed morning weighing on day d. Mean temperature and mean relative humidity were calculated over this window, and the maxima of CO, H_2_S, and NH_3_ within the same window were used as indicators of gas exposure. These summaries were indexed to the weighing day so that all environmental predictors reflected conditions that had already occurred by the time body weights were recorded and ration decisions for that day were made.

To prevent data leakage during model evaluation, all imputation and scaling parameters were estimated exclusively on the training set. Missing numerical features were imputed with the training set mean $\mu_{j}$; missing categorical features were imputed with the training set mode $m_{j}$. Each input value $x_{i,j}$ was then mapped to [0, 1] with a min-to-max scaling,

(5) $$z_{i,j}=\frac{x_{i,j}-{\;x}_{j}^{\min}}{x_{j}^{\max}\;-{\;x}_{j}^{\min}+\varepsilon },\;\;\varepsilon \;>\;0.$$

where ${\;x}_{j}^{\min}$ and $x_{j}^{\max}$ denote the minimum and maximum of feature *j* in the training set, and ε is a small positive constant to avoid division by zero.

Accordingly, the daily body-weight model used $x_{i}\mapsto z_{i}$ and the total-feed requirement model used $x_{a,r}\mapsto z_{a,r}$. The dataset was partitioned into training and test sets at a 9:1 ratio using a batch-wise temporal split based on the batch index mapping *g*(*i*). The dataset was partitioned into training and test sets at a 9:1 ratio using a batch-wise temporal split based on the batch index mapping *g*(*i*). We enforced this batch-wise temporal split and further validated the models with repeated blocked cross validation (CV, k = 5) to estimate the variance of the coefficient of determination (R^2^), root mean squared error (RMSE), mean absolute error (MAE), and mean squared error (MSE).

**Figure 2:**
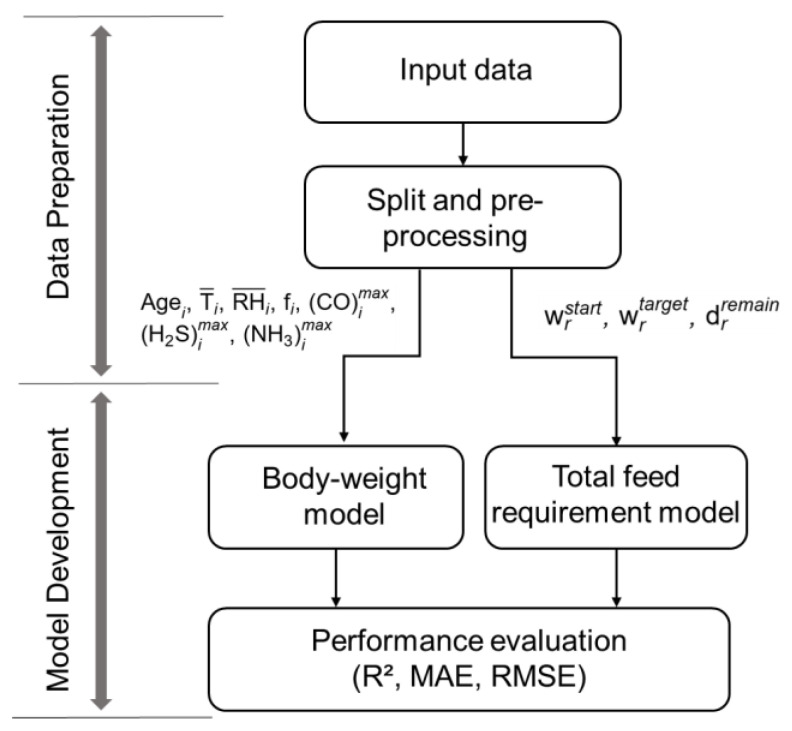
Workflow for preprocessing and model training. Models are evaluated by R^2^, MAE, and RMSE.

Breed-specific weight models were trained independently for each breed using RF, XGBoost, ET, and ANN. The overall preprocessing, temporal partitioning based on g(i), and model training workflow are summarized in Figure 2. Hyperparameters for the tree-based models were tuned within predefined search ranges, including 100–600 trees and maximum depth 3–10 for RF and ET, and learning rates 0.01–0.10 and 200–800 trees for XGBoost. The final hyperparameter settings reported in Table 1 correspond to the best-performing configurations identified through repeated blocked cross validation (CV, k = 5). Model selection was based on mean RMSE across folds, with MAE and R^2^ serving as secondary criteria, while MSE was monitored as the squared error loss. To ensure reproducibility and capture variability, a fixed random seed was applied to all tree-based models, and the ANN was trained under five independent initializations. Variability across the blocked CV folds and ANN initializations was reflected in the reported standard deviations and 95% confidence intervals for R^2^, RMSE, and MAE.

**Figure 3:**
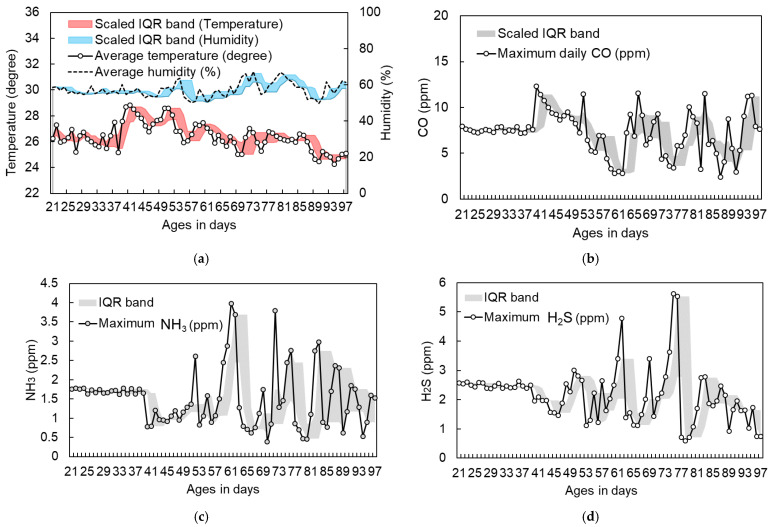
Temporal profiles of environmental variables during the training period, with shaded interquartile range (IQR) bands summarizing within-day variability. (**a**) Daily average temperature (°C, solid line) and average relative humidity (percent, dashed line) with scaled IQR bands for temperature (red shading) and humidity (blue shading). (**b**) Daily maximum CO concentration (ppm, open circles) with a scaled IQR band (grey shading). (**c**) Daily maximum NH_3_ concentration (ppm, open circles) with IQR band. (**d**) Daily maximum H_2_S concentration (ppm, open circles) with IQR band. These summaries provide context for the feature importance analysis by indicating both the central tendency and short-term dispersion of environmental covariates across the rearing period.

**Figure 4:**
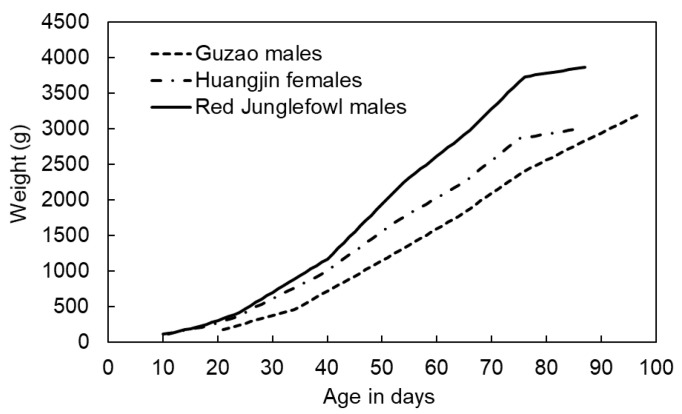
Average cage-level body-weight trajectories of the three indigenous breeds in the training period.

To contextualize the training data used for model development, Figure 3 presents the daily environmental profiles together with shaded interquartile range (IQR) bands that summarize the within-day variability of temperature, relative humidity, CO, NH_3_, and H_2_S. These unit-scaled curves and IQR bands provide both central tendency and dispersion, offering a clearer basis for interpreting the feature importance results in Section 3. Figure 4 displays the average cage-level body-weight trajectories of the three indigenous breeds in the training cohort, with unit scales shown to illustrate growth patterns over the rearing period at the cage level.

### 2.3. Machine Learning Models

This section summarizes the supervised regressors used for cage-level daily live-weight prediction and interval total-feed estimation. We formalize the model families, RF, XGBoost, ET, and an ANN, together with notation for inputs and outputs.

#### 2.3.1. RF

An RF model is composed of multiple decision trees, and the final prediction is obtained by averaging the outputs of all trees [34]:

(6) $${\hat{y}}_{i}^{\mathrm{RF}}=\frac{1}{M_{\mathrm{RF}}}\sum_{m=1}^{M_{\mathrm{RF}}}T_{m}(z_{i};\;\Theta_{m})$$

where $M_{\mathrm{RF}}$ denotes the number of trees, and $\Theta_{m}$ denotes the split parameters for the m-th tree $T_{m}$. For regression, the prediction at a terminal (leaf) node is typically computed as the mean of the training responses that fall into that node. Randomness in feature selection and split points is injected during training to improve robustness and reduce overfitting.

#### 2.3.2. XGBoost

XGBoost is an ensemble method based on gradient boosting decision trees (GBDT) and belongs to the boosting family [35,36]. It can capture strong nonlinear relationships and handle large-scale datasets efficiently. Training proceeds from an initial prediction (often the training set mean) and adds one regression tree per boosting round to fit the residuals. The final prediction is the sum of all trees:

(7) $${\hat{y}}_{i}^{\mathrm{XGB}}=\sum_{t=1}^{K}f_{t}(z_{i})$$

where K is the number of trees and $f_{t}$ is the t-th regression tree. At boosting round t, the objective minimized is

(8) $${\mathrm{Obj}}^{\left(t\right)}=\sum_{i=1}^{n}\;l\left(y_{i},\;{\hat{y}}_{i}^{(t-1)}+f_{t}(z_{i})\right)\;+\;\Omega \left(f_{t}\right)$$

where l(⋅,⋅) is the loss function and Ω(⋅) is a regularization term that penalizes tree complexity in order to improve generalization. Feature importance is commonly quantified by the cumulative reduction in loss or impurity attributable to splits on each feature.

#### 2.3.3. ET

The ET model is an averaging ensemble of randomized regression trees [37]. It is similar to a random forest, but at each node both the candidate feature subset and the split thresholds are drawn at random, which increases training speed and decorrelates trees. The ensemble prediction is computed as

(9) $${\hat{y}}^{\mathrm{ET}}=\frac{1}{M_{\mathrm{ET}}}\sum_{m=1}^{M_{\mathrm{ET}}}\rho_{m}(z;\;\Phi_{m}),$$

where $M_{\mathrm{ET}}$ is the number of trees, $\rho_{m}$ is the m-th tree, $\Phi_{m}$ collects its random partition parameters, and z denotes the scaled input vector (instantiated as $z_{i}$ for daily-weight prediction and $z_{a,r}$ for total-feed prediction).

During training, the contribution of each feature to splits can be measured using impurity reduction. Let D denote the sample set at a node and $\{D_{i}{\}}_{j}$ the child subsets obtained by splitting on feature j. The impurity reduction is defined as

(10) $$\Delta I(\mathrm{feature}\;j)\;=\;I(D)-\sum_{i}\frac{\left|D_{i}\right|}{\left|D\right|}\;I(D_{i}),$$

where |⋅| denotes the number of samples and I(⋅) is a node-impurity measure. For regression trees, a common choice is the within-node variance:

(11) $$I(D)=\frac{1}{\left|D\right|}\sum_{(z,y)\in D}(y\;-\;{\bar{y}}_{D}{)}^{2},\;\;{\bar{y}}_{D}=\frac{1}{\left|D\right|}\sum_{(z,y)\in D}y.$$

Splitting continues until stopping criteria, such as minimum node size or maximum depth, are satisfied.

#### 2.3.4. ANN

A feedforward artificial neural network was implemented as a multilayer perceptron for scalar regression from the input vector z to body weight. The model defines a parametric mapping [38]

(12) $${\hat{y}}_{i}^{\mathrm{ANN}}\;=\;g_{\Psi }(z_{i}),$$

where $\Psi \;=\;\{W^{\left(l\right)},\;b^{\left(l\right)}{\}}_{l=1}^{L}$ denotes the full set of ANN parameters, with $W^{\left(l\right)}$ the weight matrix of layer l and $b^{\left(l\right)}$ the corresponding bias value. With L−1 hidden layers, the forward propagation is

(13) $$h^{\left(1\right)}=\sigma (W^{\left(1\right)}z_{i}+b^{\left(1\right)}),\;h^{\left(l\right)}=\sigma (W^{\left(l\right)}h^{(l-1)}+{\;b}^{\left(l\right)})\;(l=2,\ldots ,\;L-1),$$

with rectified linear activation σ(⋅) in hidden layers and a linear output for continuous targets. The scalar output layer is

(14) $${\hat{y}}_{i}^{\mathrm{ANN}}=W^{\left(L\right)}h^{(L-1)}+b^{\left(L\right)},$$

Training minimizes the mean squared error with an $L_{2}\text{-}\mathrm{norm}$ weight penalty, expressed as follows:

(15) $$\mathrm{Obj}\;=\;\frac{1}{n}\sum_{i=1}^{n}(y_{i}\;-\;{\hat{y}}_{i}^{\mathrm{ANN}}{)}^{2}+\lambda \sum_{l=1}^{L}{\left\|W^{\left(l\right)}\right\|}_{2}^{2},$$

where ${\left\|\cdot \right\|}_{2}$ denotes the $L_{2}\text{-}\mathrm{norm}$ operation and λ is the regularization coefficient. The model was optimized using the Adam algorithm with mini-batches of size B, learning rate η, and up to E training epochs. Early stopping was applied based on validation loss with patience p. Regularization included dropout with rate d, whereon d denotes the dropout probability applied to the hidden layers, and batch normalization was used to stabilize layer statistics. Inputs z are standardized using training set moments to ensure comparable feature scales.

### 2.4. Feeding Schedule Algorithm

The feeding schedule procedure operates as an offline decision-support tool within the simulation framework rather than as a real-time controller. Its purpose is to distribute the predicted interval-level feed amount across days for simulation purposes. Let interval indices be $r\;=\;1,\ldots ,{\;n}_{b}$, and and let the set of daily indices belonging to interval r be $I_{r}\subset \{1,\ldots ,\;n\}$ with cardinality $|I_{r}|\;=\;d_{r}^{\mathrm{remain}}$. The single-bird total feed for interval r is predicted by the total-feed model $M_{F}(▪)$

(16) $${\hat{F}}_{r}=M_{F}\left(w_{r}^{\mathrm{start}},\;w_{r}^{\mathrm{target}},\;d_{r}^{\mathrm{remain}}\right).\;$$

If historical intake curves are available, obtain non-negative, unnormalized daily weights $\{\pi_{r,i}{\}}_{i\in I_{r}}$ and normalize them to sum to one:

(17) $${\tilde{\pi }}_{r,i}=\frac{\pi_{r,i}}{\sum_{j\in I_{r}}\pi_{r,j}},\;\;f_{r,i}\;=\;{\hat{F}}_{r}\;{\tilde{\pi }}_{r,i},\;i\;\in \;I_{r}.$$

If such data are unavailable, use an arithmetic progression. Let $m_{i}$ denote the position of day i within interval $I_{r}$. Define

(18) $$f_{r,i}^{\mathrm{raw}}={\;a}_{r}+(m_{i}-\;1)d_{r},\;\;i\;\in \;I_{r},$$

where $a_{r}$ and $d_{r}$ are the first term and the common difference, respectively. Scale the sequence to match the interval total:

(19) $$f_{r,i}={\hat{F}}_{r}\frac{f_{r,i}^{\mathrm{raw}}}{\sum_{i\in I_{r}}f_{r,i}^{\mathrm{raw}}},$$

Intuitively, use the historical curve when available, otherwise a linearly increasing allocation, while preserving the interval total. Map the interval allocation back to day-level notation by $f_{i}:=f_{r,i}$ for $i\;\in {\;I}_{r}$, and compute the cage-level daily ration using the pen mapping g(i) and the pen size $N_{g(i)}$:

(20) $$F_{i}:=N_{g(i)}\;f_{i},\;\;i\;=\;1,\ldots ,n$$

Using the previously defined ${\mathrm{Age}}_{i}$ and environmental aggregates ${\bar{T}}_{i},{\;\bar{\mathrm{RH}}}_{i},\;(\mathrm{CO}{)}_{i}^{\max},\;(H_{2}S{)}_{i}^{\max},\;(NH_{3}{)}_{i}^{\max}$, replace the daily feed input by $f_{r,i}$ and evaluate the daily body-weight model ${\;M}_{w}(▪)$:

(21) $${\hat{y}}_{i}\;={\;M}_{w}({\mathrm{Age}}_{i},{\;e}_{i}\;,\;f_{r,i}),\;\;i\;\in \;I_{r}$$

where ${\;e}_{i}\;=\;({\bar{T}}_{i},{\;\bar{\mathrm{RH}}}_{i},\;(\mathrm{CO}{)}_{i}^{\max},\;(H_{2}S{)}_{i}^{\max},\;(NH_{3}{)}_{i}^{\max})$ denotes the vector of environmental predictors for day i. For practicality, daily gains

(22) $${\hat{y}}_{i}\;-{\hat{\;y}}_{i^{-}},$$

where $i^{-}$ denotes the previous day in $I_{r}$, may be softly clipped to a reasonable band. If the simulated terminal weight at the end of $I_{r}$ deviates from target weight $w_{r}^{target}$, the interval total feed is updated proportionally:

(23) $$F_{r}^{(j+1)\;}={\;F}_{r}^{\left(j\right)}\cdot \frac{w_{r}^{\mathrm{target}}}{{\hat{w}}_{r,\mathrm{end}}^{\left(j\right)}},$$

until $|{\hat{w}}_{r,end}^{\left(j\right)}-w_{r}^{\mathrm{target}}|\;\leq \;\epsilon$ or $j\;\geq \;J_{\max}$. Intuitively, if increased total feed produces a higher terminal weight, this proportional update nudges the predicted trajectory toward the target. The interval allocation satisfies

(24) $$\sum_{i\in I_{r}}f_{r,i}=F_{r},$$

and the interval-level FCR is

(25) $${\mathrm{FCR}}_{r}=\frac{\sum_{i\in I_{r}}f_{r,i}}{\;\;w_{r}^{\mathrm{target}}-{\;w}_{r}^{\mathrm{start}}\;}.$$

The interval total-feed model was implemented using ET, software versions are reported in Section 2.5.4. Given the current pen-average live weight per bird $w_{r}^{\mathrm{start}}$, the target weight $w_{r}^{\mathrm{target}}$, and the remaining number of days $d_{r}^{\mathrm{remain}}$, the model estimates the total feed required per bird for the remainder of the rearing period. When available, historical daily intake profiles are used to shape the provisional day-by-day allocation; otherwise, a simple arithmetic profile is applied.

**Figure 5:**
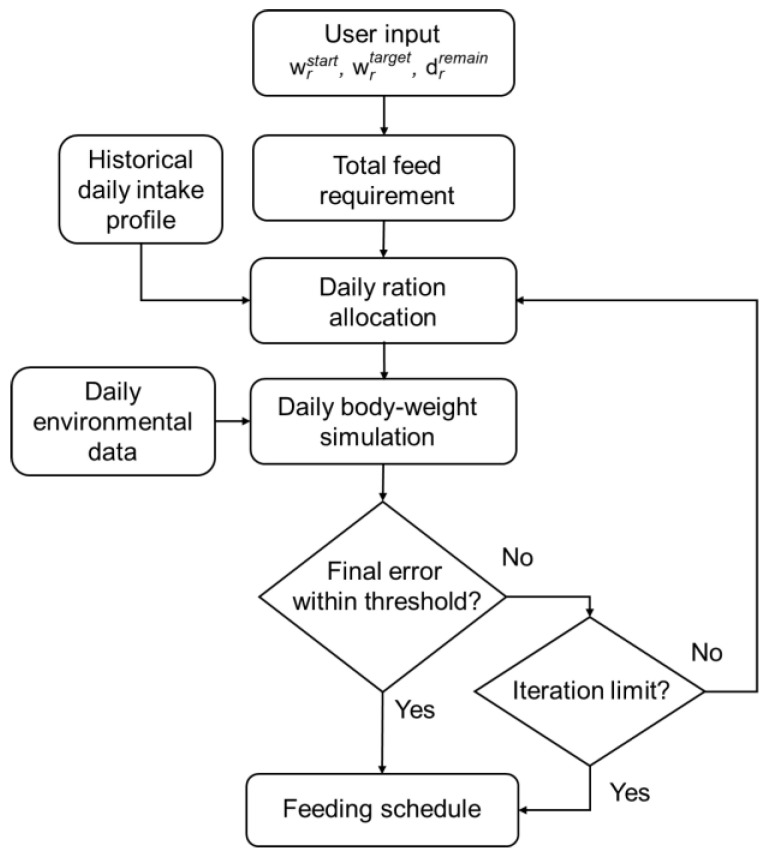
Workflow for daily ration allocation and daily body-weight simulation. Side inputs include historical intake profiles and daily environmental data. The loop terminates when the terminal error is within a predefined tolerance or the iteration limit is reached.

Operational constraints on lower and upper bounds and day-to-day changes are enforced while keeping the interval total fixed. Based on prior feeding management guidelines and observed feeding responses in indigenous chickens, this range was set at approximately ±10% of the previous day’s ration. Daily environmental records are incorporated into the body-weight simulation, after which the terminal error relative to the target is checked. If the error exceeds the tolerance, the interval total feed is proportionally adjusted and the steps are repeated until the tolerance is met or the iteration limit is reached (see Figure 5).

### 2.5. Animals, Housing, and Experimental Design

#### 2.5.1. Poultry Data

**Figure 6:**
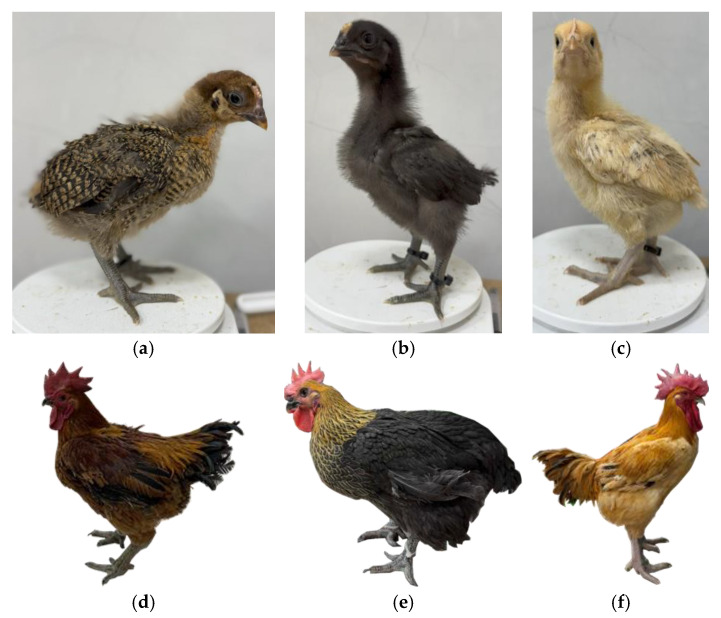
Representative phenotypes of three Taiwanese native chicken types at chick and adult stages: (**a**) Guzao male chick, (**b**) Huangjin female chick, (**c**) Red Junglefowl male chick; (**d**) adult Guzao male, (**e**) adult Huangjin female, (**f**) adult Red Junglefowl male.

This work was conducted from 5 November 2024 to 20 January 2025 in an indoor poultry production facility operating under routine commercial husbandry practices. Three native Taiwanese types were studied: Guzao males, Huangjin females, and Red Junglefowl males. Both sexes were included to reflect the principal production classes in Taiwan’s indigenous chicken sector. Each breed and sex group was modeled and evaluated independently to account for their distinct growth and feeding characteristics. In Figure 6a,d, Guzao males display a bright red single comb; chicks show mixed black and brown plumage, and adults carry predominantly brown to dark brown body feathers with darker tail coverts. In Figure 6b,e, Huangjin females are charcoal black as chicks and, as adults, retain black body plumage with distinctly golden neck hackles. In Figure 6c,f, Red Junglefowl males exhibit mottled, barred brown chick plumage and golden to orange adult plumage with red or golden hackles and darker tails.

Under conventional husbandry, these types are typically marketed at approximately 12 to 15 weeks of age, with target live weights defined by product specifications. Guzao males are usually lighter (about 2.0 kg), whereas Huangjin females and Red Junglefowl males are often finished to heavier weights to meet fresh-market preferences. Four birds of a single breed were housed per cage, and the cage was treated as the experimental unit.

#### 2.5.2. Housing and Husbandry

**Figure 7:**
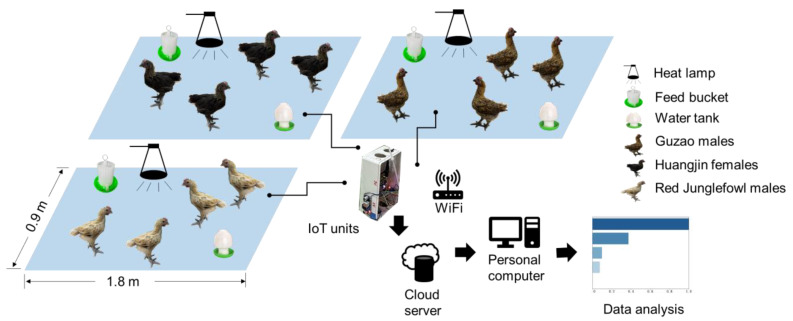
Facility layout and group allocation in the indoor test area. Three cages were installed in one room for Guzao males, Huangjin females, and Red Junglefowl males, four chickens per cage. Sensor heads were placed 30 cm above the floor near feeders and drinkers.

Three metal cages were installed in a single room, with one cage per breed and four birds per cage, as shown in Figure 7. Each cage was placed on a tiled floor with disposable paper litter and was equipped with one feeder and one ball-type drinker. During brooding, a heat lamp was suspended above each cage to maintain thermal comfort. The lighting schedule, light intensity, and ventilation settings were predetermined and checked using a lux meter and an anemometer to keep conditions consistent across cages.

A single commercial feed with a fixed formula was used throughout. In this trial the feed formulation was held constant, and the scheduling system adjusted only the daily ration quantity rather than the nutritional composition. Feeding was ad libitum, with scheduled replenishment at 08:00 and 16:00 to avoid overnight depletion. Water was available at all times through ball drinkers. Reservoirs were checked at 08:00, 14:00, and 20:00 and refilled as needed. Feeders and drinkers were cleaned daily with disinfectant prepared to 1% Virkon S, kept in contact for ten minutes, rinsed with potable water, and air dried. A deep clean was performed weekly after manual scrubbing. Litter was spot cleaned twice per day and completely replaced every seven days. Excreta and used litter were placed in double bags and removed from the room within two hours in accordance with biosafety rules. Birds were inspected once daily at a fixed time, and health logs recorded behavior, movement, and fecal traits. All procedures followed institutional guidance on animal welfare and biosecurity.

#### 2.5.3. Measurements

Within each breed, birds were randomly drawn from the supplier cohort and assigned to the study cage. No between-cage replication was implemented within breed. To reduce time-of-day bias, the weighing order was rotated weekly across cages. Masking was not feasible because breed and cage were visually apparent; data entry and preprocessing used coded identifiers. All birds were weighed on a calibrated electronic scale, and initial weights were verified to fall within ±5% of the overall mean. Daily cage feed intake was computed as feed offered minus feed refusals, corrected for spillage. Spillage was collected in removable trays placed under feeders and weighed at day end. At every weighing session, all four birds in each cage were weighed individually using a calibrated electronic scale, after which the cage-level mean was computed and used for modeling.

Environmental variables were monitored for temperature, relative humidity, and the concentrations of CO, H_2_S, and NH_3_ using a temperature and relative humidity sensor (Model: DHT22; Aosong Electronics Co., Ltd., Guangzhou, China), a CO sensor module (Model: MQ-9; Henan Hanwei Electronics Co., Ltd., Zhengzhou, China), an H_2_S sensor module (Model: MQ-136; Huaban Electronics Co., Ltd., Shenzhen, China), and an NH_3_ sensor module (Model: MQ-137; Hanwei Electronics Co., Ltd., Zhengzhou, China). Each sensor head was mounted at a fixed height of thirty centimeters above the floor and positioned away from heat lamps and ventilation inlets. All sensors were calibrated before the trial. An ESP32-based microcontroller (Model: ESP32-WROOM-E/UE; Espressif Systems Co., Ltd., Shanghai, China) handled acquisition with a sampling interval in seconds. Data were streamed to a ThingSpeak cloud server (MathWorks; Natick, MA, USA) through the message queuing telemetry transport (MQTT) protocol and written concurrently to a local microSD card. During outages, data were cached locally and backfilled after connectivity was restored. Both cloud and local logs included cage identifiers, timestamps, temperature, relative humidity, and gas concentrations. Figure 7 presents the experimental layout of three cages, each housing one breed with four chickens per cage. Each cage was equipped with a feeder and a ball-type drinker. The environmental data were collected with an IoT data acquisition unit and transmitted to a cloud server over WiFi. The cloud data were then available for download to a local computer for visualization and analysis. The upper left panel shows the Huangjin female cage, the upper right panel shows the Guzao male cage, and the lower left panel shows the Red Junglefowl male cage.

#### 2.5.4. Hyperparameter Settings

Models were trained in Python 3.9.18 using Spyder 5.5.1. The software stack comprised scikit learn 1.4.1.post1, XGBoost 2.1.4, pandas 2.2.1, NumPy 1.23.5, and Matplotlib 3.8.3. All experiments were executed on a workstation with an Intel Core i7 12700 CPU, an NVIDIA GeForce RTX 3080 Ti GPU, and 16 GB of RAM. Hyperparameter settings are reported in Table 1.

The architecture of ANN consists of one input, two hidden (L=3) and one output layer. Hidden layers used rectified linear activation function σ(⋅)=ReLU with widths 64 and 32; the output layer was linear for scalar regression. Optimization used ‘Adam’ algorithm with initial learning rate η = 0.001. Weight decay was applied through $L_{2}\text{-}$norm regularization with penalty λ=0.001. Training proceeded up to E = 2000 epochs with convergence tolerance $10^{-4}$. Early stopping monitored a held-out validation split of 0.1 with patience p = 20 epochs without improvement. A fixed random seed ensured reproducibility. Inputs z contained seven covariates per day: ${\mathrm{Age}}_{i},\;\;\;{\bar{T}}_{i},\;{\bar{\mathrm{RH}}}_{i},\;$ $f_{i},\;(\mathrm{CO}{)}_{i}^{\max},\;(H_{2}S{)}_{i}^{\max},$ and $(NH_{3}{)}_{i}^{\max}$.

## 3. Results

### 3.1. Performance of Body-Weight Model

We compared the predictive performance of four models, RF, XGBoost, ET, and ANN, across three indigenous chicken breeds.

A total of 231 daily cage-level records were collected over 77 days for each breed. Using the batch index mapping *g*(*i*), 207 records were allocated to the training subset and 24 records were reserved for testing per breed. The temporal partitioning followed the batch-wise procedure described in the Materials and Methods section.

As shown in Table 2, ET achieved the best performance. For Huangjin females, Guzao males, and Red Junglefowl males, RMSEs were 21.59 g, 20.37 g, and 45.94 g, with corresponding R^2^ values of 0.9995, 0.9995, and 0.9987. XGBoost ranked second, with RMSEs of about 47.08 to 61.18 g and R^2^ values of 0.9969 to 0.9977. RF was weaker, with RMSEs of 53.64 to 80.08 g and R^2^ values of 0.9935 to 0.9970. The larger RMSE for Red Junglefowl males suggests faster growth and greater within-breed variability, which increases prediction difficulty. Although the tree-based models achieved extremely high in-sample R^2^ values, these results should be interpreted with caution. Each breed contributed only 207 training records derived from 12 birds, and the smooth and monotonic structure of daily weight curves can allow ensemble trees to closely memorize the observed trajectories.

### 3.2. Environmental Associations with Body Weight

**Figure 8:**
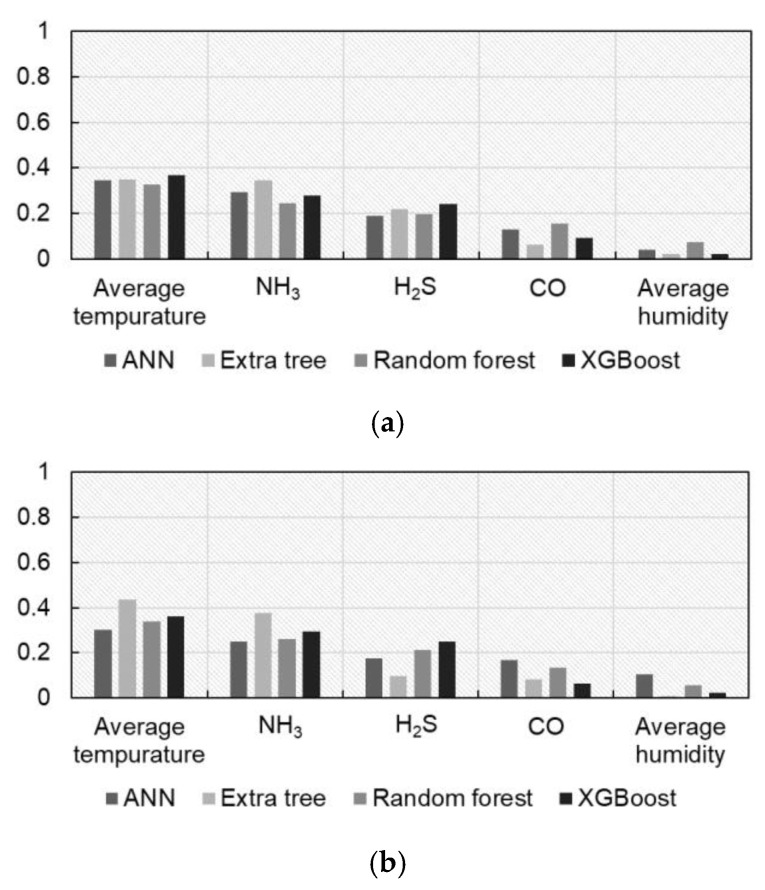

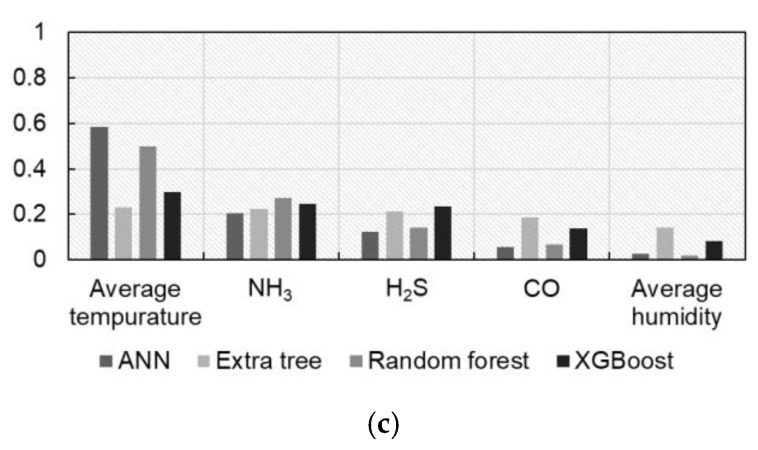
Feature analysis of machine learning models for three chicken breeds. (**a**) Guzao males; (**b**) Huangjin females; (**c**) Red Junglefowl males.

Environmental variables were evaluated for their relative contributions to body-weight prediction across models and breeds (Figure 8). In the model-specific analyses, temperature generally showed the strongest association, but the ranking of gas-related covariates differed across breeds. Ammonia contributed most strongly for Huangjin females, whereas hydrogen sulfide and carbon monoxide were more prominent in Red Junglefowl males and Guzao males, respectively. These breed-specific differences reflect heterogeneous environmental sensitivities as well as the limited number of independent units per breed.

To obtain a unified comparison based on a common feature set, environmental-only Extra Trees models were fitted separately for each breed, and SHapley Additive exPlanations (SHAP) values were computed [39]. When averaged across breeds, mean absolute SHAP values were highest for temperature and carbon monoxide, intermediate for hydrogen sulfide and ammonia, and lowest for relative humidity (Table 3). IQR quantified uncertainty across samples. Relative humidity exhibited the narrowest IQR, consistent with its minimal overall contribution. Ammonia showed moderate IQR values that partially overlapped with those of hydrogen sulfide, whereas hydrogen sulfide had a larger IQR and a higher mean SHAP value than ammonia. Taken together, the aggregated ordering across breeds is summarized as temperature and carbon monoxide contributing most strongly, followed by hydrogen sulfide and ammonia, with relative humidity contributing least.

### 3.3. Validation of the Feeding Schedule

The scheduling procedure was validated on the second batch of indigenous chickens. Pre-experimental rearing occurred at the supplier’s farm from hatch to approximately day 46 under routine husbandry. Birds had ad libitum access to water and commercial starter then grower diets; housing used naturally ventilated chickens with litter, routine cleaning, and no deliberate thermal or ammonia challenges. No medications were administered for research purposes. Detailed records on diet formulation, exact stocking density, and environmental measurements before day 46 were not available for audit, and complete uniformity across breeds could not be confirmed. Four Guzao males had a mean body weight of 785 g on Day 46 with a target of 2500 g on Day 100. The schedule was generated with the ET method, which provided the best performance for daily body-weight estimation and interval total-feed requirement estimation (hyper-parameter: 300 trees, maximum depth 6, minimum samples per split 2).

**Figure 9:**
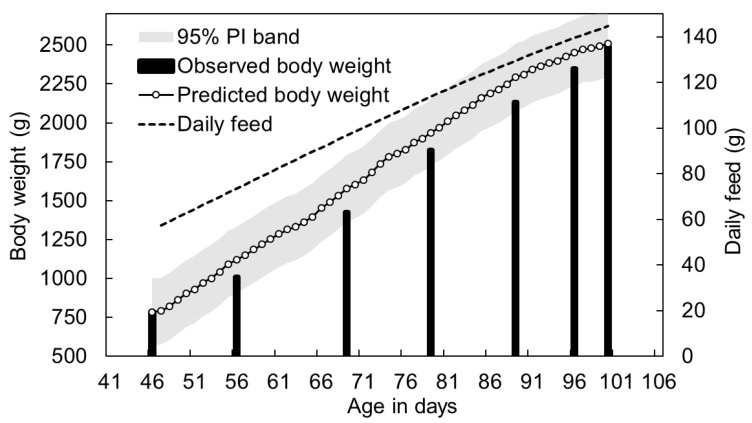
Predicted and observed body-weight trajectory with scheduled daily feed for Guzao males. Open circles indicate predicted daily body weight, solid bars represent observed weights on measurement days, and the dashed line denotes the planned daily ration. The shaded region corresponds to the 95% prediction interval (PI) band. Annotated values summarize predictive accuracy over the evaluation period (MAE = 93.57 g, RMSE = 109.51 g, R^2^ = 0.9682).

First, the interval total-feed requirement per chicken from the current day to the target day was estimated. The total was then distributed across days using a historical proportion curve under a mass conservation constraint, with bounds on the daily minimum, daily maximum, and day-to-day variation to form a preliminary per-chicken daily ration. Next, the daily ration together with same day environmental variables served as inputs to the daily body-weight model to simulate growth forward in time. During the validation phase, the predicted daily body-weight trajectory closely followed the observed growth pattern of Guzao males, with scheduled rations increasing from approximately 57 g to 145 g per bird across the rearing period. Measurement-day observations at Days 46, 56, 69, 79, 89, 96, and 100 recorded mean body weights of 785, 1009, 1425, 1825, 2134, 2350, and 2490 g, respectively, against which model predictions achieved MAE = 93.57 g, RMSE = 109.51 g, and R^2^ = 0.9682. These values are substantially higher than the in-sample RMSE of 20–46 g reported in Table 2, which is expected because the validation analysis reflects performance under a different production cycle. To visualize the agreement between predicted and observed trajectories, Figure 9 presents the daily predictions with a 95% prediction interval (PI) band, alongside the planned daily ration and the observed body weights on measurement days.

### 3.4. Comparison of FCR

To assess the impact of scheduling strategies on feed conversion, we compared ad libitum feeding with a model-assisted schedule. In the ad libitum group (Days 46–96), the per-chicken total-feed intake was 7506.75 g, and body weight increased from 972.5 g to 3167.25 g, yielding a gain of 2194.75 g and an FCR of 3.42. In the model-assisted group (Days 47–100), the initial weight was set to 785 g (Day 46), the per-bird (Guzao males) total-feed intake was 5578.08 g, and body weight increased from 785 g to 2490 g, yielding a gain of 1705 g and an FCR of 3.27. Relative to ad libitum feeding, the model-assisted schedule reduced FCR by approximately 4.39 percent, indicating that precise allocation curbs unnecessary feed consumption and improves economic efficiency (Table 4). The feeding trial was a single-pen pilot demonstration; therefore, the FCR results represent descriptive contrasts rather than significance-tested differences. The slight difference in observation windows (Days 46–96 vs. 47–100) reflected on-farm recording logistics and corresponds to the same growth phase of the same batch. Under these matched conditions, the observed FCR reduction remains informative as a feasibility indicator. Note: The FCR comparison in Table 3 reports Guzao males only, because the feeding schedule experiment was run on this breed as a controlled pilot; other breeds were used for model training and evaluation, not for the feeding trial.

### 3.5. Economic Analysis

A partial-budget framework was used to illustrate how technical outcomes may translate into batch-level feed cost differences. Let N be the number of birds per batch, $W_{g}$ the live-weight gain per bird (kg), $\Delta \mathrm{FCR}\;={\;\mathrm{FCR}}_{0}\;-{\mathrm{FCR}}_{1}$ the observed improvement in FCR, and $P_{f}$ the feed price (NTD·kg^−1^). The feed savings and their monetary value are

$$\Delta \mathrm{Feed}\;=\;\Delta \mathrm{FCR}\;W_{g}N,\;{\mathrm{Saving}}_{\mathrm{feed}}=\Delta \mathrm{Feed}P_{f}.$$

- Example 1: Small validation batch (four chickens, 54 days)

In the validation batch, ΔFCR = 0.15 (from 3.42 to 3.27). The observed average live-weight gain was $W_{g}=1.705$ kg, which yields ($P_{f}\;=\;14$ NTD/kg):

$$\Delta \mathrm{Feed}\;=\;0.15\times 1.705\times 4\;=\;1.02\;\mathrm{kg},\;{\mathrm{Saving}}_{\mathrm{feed}}\approx \;1.02\times 14\;=\;14.3\;\mathrm{NTD}.$$

Weekly manual weighing requires about one minute per bird, so the 54-day period entails about 32 min of labor; at 300 NTD·h^−1^ this corresponds to roughly 160 NTD. A handheld scale priced at 2000 NTD amortized over ten cycles adds approximately 200 NTD per cycle. At this small scale, feed savings fall well below labor and capital costs, so the economic signal is illustrative only rather than representative of commercial conditions.

b. Example 2: Commercial-scale illustration (N = 1000)

Using the same ΔFCR = 0.15, $W_{g}=1.705$ kg, and $P_{f}\;=\;14$ NTD/kg:

$$\Delta \mathrm{Feed}\;=\;0.15\times 1.705\times 1000\;=\;225.7\;\mathrm{kg},\;{\mathrm{Saving}}_{\mathrm{feed}}\approx \;225.7\times 14\;=\;3580\;\mathrm{NTD}.$$

If weekly sampling of 60 birds is used to calibrate the ration schedule, labor requires approximately eight hours per cycle (about 2400 NTD). Including scale amortization and routine maintenance, total measurement cost can plausibly be contained within 3000–4000 NTD per cycle. Whether net savings are positive depends on site-specific values of $P_{f}$, ΔFCR, $W_{g}$, N, and the degree of automation. Feed savings scale linearly with N, whereas labor inputs may be reduced through sampling strategies or automated weighing. These estimates are illustrative and based on a single, small validation batch. A complete economic evaluation would require multi-batch datasets and explicit consideration of depreciation beyond small equipment, as well as potential effects on mortality, carcass traits, or other production outcomes.

## 4. Discussion

### 4.1. Comparative Model Performance

This study demonstrates the feasibility of a machine learning–assisted feeding scheduler for indigenous chickens, with growth performance maintained in the validation batch. In a 54-day observational comparison involving four Guzao males reared under identical conditions, the scheduled batch exhibited an FCR of 3.27, whereas a separate ad libitum batch recorded an FCR of 3.42. Because the batches were non-concurrent and unreplicated, these values represent an observational contrast rather than evidence of biological improvement. The direction of this contrast is consistent with findings from broiler precision-nutrition studies that adjust daily intake using blended diets or automated monitoring systems [40,41,42,43], but no causal inference can be made from the present data. Growth performance, defined as final live weight and average daily gain, was maintained, consistent with reports that precision feeding can improve production efficiency [19,44]. Because the available dataset contained a modest number of cage-level observations, the high in-sample R^2^ values for tree-based models should be interpreted cautiously, and additional batches will be required to fully assess out-of-sample generalizability.

In comparative modeling, the artificial neural network yielded higher RMSE and lower R^2^ than the ensemble tree models. This outcome is expected given the characteristics of the present dataset, which contains a limited number of daily observations per breed, relatively narrow variability in several predictors, and heterogeneous feature scales. Under such conditions, feed-forward networks often exhibit sensitivity to sample size, feature distribution, and initialization, even with normalization and tuning, whereas tree-based ensembles tend to provide more stable performance in low-to-moderate-sized structured datasets [5,9,45]. The observed ANN behavior therefore reflects the inherent data regime rather than limitations of the modeling pipeline.

### 4.2. Environmental Associations

The environmental ranges observed in this study were modest (Figure 3). Across models and breeds, the ranking of environmental associations was broadly consistent, with temperature most prominent. This is aligned with evidence that higher thermal load correlates with reduced feed intake and slower growth [46,47,48]. The SHAP analysis indicated that the relative contributions of environmental variables differed across breeds. In the aggregate assessment based on environmental-only ET models, temperature and carbon monoxide showed the largest overall contributions, followed by hydrogen sulfide and ammonia, whereas relative humidity exhibited the smallest contribution (Table 3). This ordering reflects the breed-balanced means, where temperature and carbon monoxide consistently ranked highest, hydrogen sulfide and ammonia occupied intermediate positions with notable breed-specific variation, and relative humidity was lowest.

During the rearing period, daily maximum NH_3_ concentrations ranged from approximately 0.3 to 4.0 ppm, and daily maximum H_2_S concentrations from approximately 0.2 to 4.5 ppm; both ranges were far below commonly cited thresholds associateed with growth depression or respiratory irritation (NH_3_ > 25–50 ppm; H_2_S > 20–50 ppm). These low exposure levels help explain their comparatively smaller influence on predicted weight dynamics [49,50,51]. H_2_S at low ppm can trigger inflammatory and oxidative responses that impair growth, consistent with its moderate but nontrivial contribution in the model-based importance ranking [52,53]. CO served mainly as a safety indicator; concentrations were near zero, in line with guidance that CO should remain negligible and that elevations are hazardous [54,55]. Under such conditions, carbon monoxide acted primarily as a safety indicator rather than a biologically meaningful driver of growth performance. Its relatively large SHAP contribution therefore reflects small but informative model-relevant variation around a consistently low baseline, rather than exposure to harmful concentrations. Carbon dioxide was not included in the ranking because the analysis focused on thermal stress and harmful gases; under standard farm conditions, carbon dioxide typically affects body weight only at substantially elevated levels. Relative humidity showed the smallest aggregate contribution, which is consistent with its temperature-dependent role in thermal load. Humidity alone contributes limited explanatory power unless temperatures approach critical thresholds or vary across a broad range [46].

The external validity of these findings is constrained by the study design. Validation was conducted using a single follow-up batch raised in the same facility under consistent management, so performance under other seasons, production environments, or sex combinations was not assessed. The birds entered the trial at approximately day 46 under routine commercial management, and early-life conditions such as brooding temperature, stocking density, and initial diet were not standardized across breeds. These contextual considerations are important for interpretation and motivate ongoing plans to extend the evaluation across multiple batches and farm sites to assess robustness under broader production conditions.

### 4.3. Limitations and Future Work

The training and evaluation of this model were based on cage-level mean body weight and cage-level feed intake; therefore, the small effective sample size may inflate the apparent R^2^ values. Furthermore, this experiment involved mixed-sex chickens, and the potential effects of sex on growth, feed conversion ratio, and environmental responses require further validation. Although the cage experiment lacked replication, uncontrolled environmental variation was unlikely to have influenced the observed outcomes. All cages were located in the same enclosed room, with uniform ventilation, lighting, and husbandry procedures. Continuous sensor recordings confirmed that temperature, humidity, and gas concentrations remained within narrow ranges throughout the experiment, with no meaningful differences between cages. No equipment malfunctions, management inconsistencies, or localized disturbances that could systematically bias one cage relative to another were recorded. These controls reduce the likelihood that microenvironmental variation affected growth outcomes. While single-replicate experiments are limiting, they become a serious concern primarily when substantial within-treatment environmental variability is present, which was not the case in this study. Future research will be extended across additional batches, farms, and seasons to assess generalizability [56,57,58]. In addition, future work will quantify labor requirements, capital costs, and feed savings under both sampling-based and automated weighing scenarios.

## 5. Conclusions

This study developed and evaluated a preliminary proof-of-concept machine learning system for daily body-weight estimation and feeding programming in local chickens. Input variables included age, environmental measurements, and feeding records from three indigenous breeds. In comparative evaluations, the Extra Trees model achieved the highest accuracy across breeds by capturing nonlinear associations among temperature, gas concentrations, feed amount, and age. The overall in-sample accuracy was high (R^2^ up to 0.9995; RMSE approximately 20 to 46 g). However, longer-term, multi-batch, or multi-cycle datasets will be required to more fully characterize model uncertainty and generalizability, and to assess calibration and interval estimates for day-ahead weight and ration predictions. While single replication is a recognized limitation, it becomes a major concern primarily when substantial within-treatment environmental variability is present. In this study, environmental conditions remained stable and uniform across cages, which reduces the likelihood that uncontrolled variability materially influenced the observed outcomes. Although the present implementation was developed for three Taiwanese indigenous breeds under specific husbandry conditions, the modeling workflow can be adapted to other genetic lines or production systems by retraining the weight-prediction and ration-allocation models on population-specific datasets prior to deployment. Such adaptation will support more accurate decision-making and may contribute to improved efficiency and sustainability in local chicken production.

## Figures and Tables

**Table 1:** Hyperparameter settings for the three machine learning models for body-weight estimation.

	Model	RF	XGBoost	ET
Hyperparameter				
Number of trees		300	600	300
Maximum depth		6	6	6
Minimum samples per split		5	N/A	2
Learning rate		N/A *	0.05	N/A

*: N/A = not applicable.

**Table 2:** Performance comparison of body-weight prediction models for three indigenous chicken breeds. All metrics reflect in-sample performance on the training batches.

Breed	Model	MSE (g^2^)	MAE (g)	RMSE (g)	R^2^
Guzao males	RF	5588.78	51.19	74.76	0.9935
	XGBoost	2778.82	47.87	52.71	0.9969
	ET	414.91	16.77	20.37	0.9995
	ANN	18,369.71	105.21	135.53	0.9796
Huangjin females	RF	2877.06	35.95	53.64	0.9970
	XGBoost	2216.25	40.65	47.08	0.9977
	ET	466.09	15.93	21.59	0.9995
	ANN	14,051.92	85.00	118.54	0.9852
Red Junglefowl males	RF	6412.44	64.11	80.08	0.9961
	XGBoost	3742.93	53.42	61.18	0.9977
	ET	2110.75	38.22	45.94	0.9987
	ANN	32,400.33	138.16	180.00	0.9804

Note: Metrics are reported separately for each breed; variability estimates (standard deviations and confidence intervals) were derived from repeated blocked cross validation as described in Section 2.2.

**Table 3:** Environmental-only Extra Trees results at the cage level, summarized by mean absolute SHAP values and IQR.

	Guzao Males		Huangjin Females		Red Junglefowl Males		Breed-Balanced (Mean)	
	SHAP	IQR	SHAP	IQR	SHAP	IQR	SHAP (Total Mean)	IQR (Total Mean)
Average temperature	1199.76	1394.49	1326.09	1611.12	1664.01	822.90	1396.62	1276.17
Carbon monoxide	1129.65	1143.96	949.77	832.46	1330.49	1118.71	1136.64	1031.71
Hydrogen sulfide	691.10	825.98	268.54	249.70	1605.55	1546.78	855.06	874.15
Mean relative humidity	671.40	594.61	252.36	233.29	715.24	778.56	546.34	535.49
Ammonia	561.87	644.45	1528.92	1380.72	253.28	269.58	781.35	764.92

**Table 4:** Comparison of feed conversion ratios for Guzao males.

Feeding Regimen	Age (Days)	Total-Feed Intake (g/Chicken)	Total Body-Weight Gain (g/Chicken)	FCR
Ad libitum	46–96	7506.75	2194.75	3.42
Scheduled feeding (ET model)	47–100	5578.08	1705.00	3.27

## Data Availability

The original contributions presented in this study are included in the article. Further inquiries can be directed to the corresponding author.

## Abbreviations

ANN	Artificial Neural Networks
ET	Extra Trees
FCR	Feed Conversion Ratio
GBDT	Gradient Boosting Decision Trees
IQRs	Interquartile Ranges
MAE	Mean Absolute Error
PI	Prediction Interval
RF	Random Forest
R^2^	Coefficient of Determination
RMSE	Root Mean Square Error
SHAP	SHapley Additive exPlanations
XGBoost	Extreme Gradient Boosting
